# A practical introduction to microbial molecular ecology through the use of isolation chips

**DOI:** 10.1002/ece3.4748

**Published:** 2018-12-11

**Authors:** Anna M. Alessi, Kelly R. Redeker, James P. J. Chong

**Affiliations:** ^1^ Department of Biology University of York York UK

**Keywords:** antibiotics, iChip, molecular microbial ecology, teaching

## Abstract

In the context of antimicrobial resistance as one of the most serious issues faced globally by health providers, we explored a practical introduction to molecular microbial ecology. We designed field work and practical experiments for third year members of a 4 year undergraduate Masters Program, in which the students employed traditional and novel isolation techniques to identify antimicrobial activities from soil dwelling microorganisms. Students gained experience in isolating DNA from complex microbial communities, amplifying 16S rRNA genes and applied richness/diversity indices as well as principal coordinate analyses to the interpretation of the data they obtained from high throughput sequencing. Our results confirmed that isolation chips facilitate the growth of a greater diversity and different species subset from the complex soil microorganism community than traditional plate spreading techniques. However, rarefaction of 16S rRNA amplicon sequencing data showed that the majority of observed species in soil remain unculturable by current methods. Based on the written reports produced by the students carrying out the work, we concluded that the described protocols are robust and informative, that these activities provide a good practical introduction to the theories and practice of molecular ecology and can be easily deployed to groups of six or more students in a cost‐effective manner.

## 1 INTRODUCTION

Antimicrobial resistance enables microorganisms to withstand the effects of a drug that was previously effective in eliminating infection by killing or limiting bacterial growth. Resistant strains propagate under antimicrobial selective pressure and resistance is spread within a population through horizontal gene transfer and replication (Wilson, 2014). The minimal survival cost of maintaining resistance genes means that once resistance is gained it is rarely lost. Resistance spreads rapidly, especially in areas such as hospitals and care centres, where antibiotics are widely used and resistant and sensitive strains can interact (Berendonk et al., 2015). Available treatments are very limited for infections caused by multidrug resistant ESKAPE (*Enterococcus faecium*, *Staphylococcus aureus*, *Klebsiella pneumoniae*, *Acinetobacter baumannii*, *Pseudomonas aeruginosa*, and *Enterococcus* species) pathogens (Boucher et al., 2009; Falagas et al., 2005; Fischbach & Walsh, 2009; Weigel et al., 2003).

The “great plate count anomaly” (Staley & Konopka, 1985), describes the difference between what is in the soil and what can be grown using traditional methods. The anomaly was revealed through the development of culture‐independent approaches such as sequencing of 16S SSU ribosomal RNA genes and demonstrated the need for novel culturing approaches to access the majority of the soil microbial population (Pace, 1997). The screening of the very small proportion of soil microorganisms that can be cultured using synthetic media under laboratory conditions has proved to be an effective way of identifying novel antimicrobial activities, mainly from the genus *Streptomyces* (Bérdy, 2005). However, this method, based on traditional culturing techniques, now provides highly diminished new discoveries. Several alternative methods of culturing a wider range of microbes have since been developed and include targeted phenotypic culturing (Browne et al., 2016), diffusion chambers (Kaeberlein, Lewis, & Epstein, 2002), encapsulation (Zengler et al., 2002) and high dilution (Rappé, Connon, Vergin, & Giovannoni, 2002) approaches.

Introducing undergraduate students to microbial ecology at a molecular level tends to occur through the delivery of lectures and information‐based, theoretical workshops. We sought to provide students with a hands‐on learning experience of this topic through a series of managed practicals that included field work, lab work, high‐throughput DNA sequencing and analysis of the resulting data. The exercise was constrained by student timetables and limited funds. We carried out this work with two groups of undergraduate students in the third year of a 4 year taught Masters course, where the course demands a two term (approximately 18 weeks) “group project” requiring no more than 1 day per week effort. The overarching goal of this project was inspired by the Small World Initiative (Davis et al., 2017) which aims to educate students in microbiology through the search for novel antimicrobial producing microorganisms and was associated with the Microbiology Society's related Antibiotics Unearthed program (https://www.microbiologysociety.org/education-outreach/antibiotics-unearthed.html).

Students were introduced to the notion that microbial antibiotic resistance could cause 10 million deaths globally per annum by 2050 (Farrar & Davies, 2016). This threat has developed through years of antibiotic misuse and overuse but also through the lack of novel antibiotic discovery and availability in the last 30 years (Blair, Webber, Baylay, Ogbolu, & Piddock, 2015; Nathan, 2004). The discovery of teixobactin in 2014 (Ling et al., 2015) described the first novel class of antimicrobial agents since 1987. This work used an isolation chip (iChip) (Nichols et al., 2010) which was an elaboration of the in situ isolation techniques such as diffusion chambers developed by Lewis and Epstein (Kaeberlein et al., 2002). Isolation chips (iChips) consist of 384 miniature diffusion chambers that allow the growth of individual microbial species with access to metabolites from their natural environment, providing an effective means of isolating novel bacterial species that might include novel antibiotic producers.

## 2 MATERIALS AND METHODS

### 2.1 Overall study strategy

**Figure 1:**
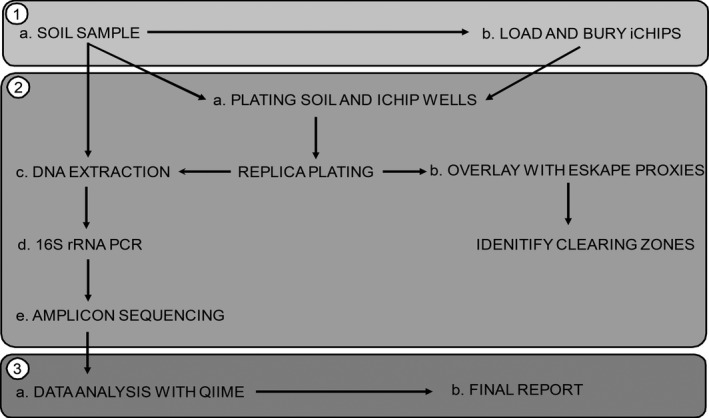
Experimental design of molecular microbial ecology group project. The 18‐week project was divided into three parts: (1) field work, (2) lab work, (3) data analysis and reporting. During fieldwork, students were provided with iChips that they loaded with soil dilutions (1a) and then buried in dedicated locations for 2 weeks (1b). Initial lab work included preparation of soil extract agar plates, plating soil dilutions, and incubated iChip wells (2a) and overlaying isolates from soil and iChips with ESKAPE indicator species (2b). Molecular work included DNA extraction directly from soil samples, and from bacterial colonies recovered from soil dilution plating and isolation chips (iChips) (2c), PCR amplification of 16S rRNA genes and electrophoretic evaluation of PCR products (2d), followed by high throughput amplicon sequencing using the MiSeq platform (2e). Outcomes of the project were assessed through data analysis using QIIME (3a) and the production of a written report (3b)

Students were provided with a series of written protocols based on Small World Initiative material (https://sites.google.com/a/york.ac.uk/chonglab/teaching) and with iChips that they loaded with soil samples collected from a variety of environmentally and biologically variable locations. The loaded iChips were reburied in the locations where the sampling occurred and allowed to incubate (Supporting Information Figure S1). While the iChips were buried, students (a) extracted DNA from the iChip sampled soil (b) produced “soil agar” plates and (c) plated a sample of this same soil on the soil agar. The iChips were recovered and the contents of individual wells were plated onto soil agar (Figure 1, Supporting Information Figure S2). DNA was separately extracted from a “traditional” cultivation approach on spread plates and iChip cultivation. All three DNA samples (from the soil, the spread plate and the iChip enrichment) were subjected to 16S SSU rRNA gene amplification by the students, who then submitted their samples for library preparation and high‐throughput sequencing (Supporting Information Table S1). The resulting data were analysed by students using QIIME (Caporaso et al., 2010) to determine the relative abundance and number of observed species. While the samples were being sequenced, the isolates propagated using iChip and traditional spread cultivation approaches were screened for antimicrobial properties using safe relatives of multidrug resistant ESKAPE pathogens (Figure 1) (Boucher et al., 2009). Students analysed their data for the preparation of assessed reports. For this work, sequencing data were combined and re‐analyzed (by AMA).

### 2.2 Study sites

Soil was collected from two locations: an arable field under onion cultivation at the time of analysis located at Hagg Farm (HF, Askham Bryan, 53°56′16″N, 1°10′10″W) and Three Hagges Wood Meadow, a previously arable agricultural site replanted as a wildflower grassland mix encompassing more and less diverse aboveground plant communities (THW, 53°50′51″N, 1°2′50″W). iChip locations were marked and GPS coordinates were noted to facilitate recovery (Supporting Information Figure S1). Sampling of the HF and THW sites took place in October 2015 and October 2016, respectively.

### 2.3 iChip loading

Soil cell density was estimated from six soil replicates that were diluted and stained with Hoescht 33,258 (50 μg/ml) and counted under a fluorescence microscope using a THOMA counting chamber. The average cell density was 2.71 × 10^8^ cells/ml (*n* = 6, *SD* = ±1.11 × 10^8^) hence a dilution factor of 3 × 10^5^ was recommended to reach a cell density of 1 × 10^3^ cells/ml, ensuring an average of one cell per iChip well (1 µl volume).

Isolation chips (Supporting Information Figure S2) were sterilized with 70% (v/v) ethanol prior to use. For the HF site, iChip loading took place in the field whereas for the THW site, iChips were loaded in the laboratory to maintain sterile conditions and prevent contamination of negative controls. To achieve the required dilution, one gram of soil was collected, added to 10 ml 1× PBS (8 g NaCl, 0.2 g KCl, 1.44 g Na_2_HPO_4_, 0.24 g KH_2_PO_4_ per 1 L, pH 7.4) shaken vigorously for 30 s then allowed to settle for 3 min. One millilitre of the suspension supernatant was transferred to a 2 ml Eppendorf tube, 10 μl of this was added to 2.99 ml 1× PBS followed by a transfer of 20 μl of the resulting mixture to 14.98 ml 1× PBS. Five millilitre of 1.4% (w/v) molten agar (45°C) was added to the final dilution and mixed thoroughly. The molten agar mix was poured immediately into a Petri dish and the central plate of an iChip was submerged. Two membranes (Whatman Nuclepore Track‐Etched, MB PC 0.05 μm, 47 mm) were used to seal wells on both sides of the central plate. The loaded, reassembled iChips were buried 10 cm below surface level at the locations indicated on the maps (Supporting Information Figure S1) (https://sites.google.com/a/york.ac.uk/chonglab/teaching). Soil temperature was recorded as 6°C (±4°C). In total 14 (two per student) iChips were buried at the HF site and six iChips were buried at the THW site, for 2 weeks.

### 2.4 Culturing

Soil collected from experimental sites was used to make soil extract. Briefly, 0.5 kg of soil was resuspended in 750 ml distilled water then autoclaved at 121°C for 15 min. The supernatant was centrifuged at 4,000 ***g*** for 20 min at room temperature then further clarified by filtration through filter paper (Whatman, 90 mm, #1001–090). Soil‐extract agar was made using 0.5 g K_2_HPO_4_, 0.1 g of glucose and 20 g of agar in one liter of soil extract.

Recovered iChips were disassembled and membranes were carefully removed from the central plate. Each agar plug was transferred onto a soil‐extract agar plate using a separate, fresh sterile metal rod. In addition to iChip plating, traditional culturing was used. One gram of soil from the same site was diluted with 1× PBS to a final concentration of 1 × 10^3^/ml bacterial cells (see above); 50 μl of this dilution was spread onto soil‐extract agar plates. All plates were incubated at room temperature for minimum of 1 week.

### 2.5 Replica plating and overlay with indicator species

Safe relatives (*Enterococcus raffinosus* ATCC 49464, *Staphylococcus epidermidis* ATCC 14990, *Escherichia coli* ATCC 11775, *Pseudomonas putida* ATCC 12633, *Enterobacter aerogenes* ATCC 51697) of ESKAPE pathogens (*E. faecium*, *S. aureus*, *K. pneumoniae*, *A. baumannii*, *P. aeruginosa* and *Enterobacter* spp.) were obtained from ATCC and grown aerobically in nutrient broth (Oxoid) at 37°C (except for *P. putida* which was grown at 26°C). Fully grown iChip plates were replica plated by picking individual colonies with a sterile tip and transferring them onto three freshly made soil‐extract agar plates. The plates were incubated at room temperature for a minimum of 1 week or until sufficient growth was obtained. One set of replica plates was overlaid with an indicator species: 100 µl of overnight culture was mixed with 7 ml of 0.75% (w/v) molten nutrient agar (45°C) and poured over the colonies. Plates were incubated at 37°C or 26°C for 3 days before being examined for clearing zones indicative of antimicrobial activity. Colonies that showed clearing zones were recovered, resuspended in 20 µl of sterile nutrient broth and streak purified on nutrient agar. A second set of replica plates was used to perform DNA extractions (see below).

### 2.6 Extraction of metagenomic DNA

DNA was extracted from soil (“soil”), colonies that grew on soil extract agar replica plates from the soil dilution spread plating (“spread”) and, iChip (“iChip”) enrichment. Colonies on soil‐extract plates were recovered by flooding each plate where colonies appeared to have fully developed with 2 ml of 1× PBS. The suspension was centrifuged at 4,000 ***g*** for 10 min at room temperature and supernatant was removed. DNA from the resulting pellet and soil was extracted using an UltraClean Microbial DNA Isolation kit (QIAGEN) and Power Soil DNA extraction kit (QIAGEN), respectively, following the manufacturer's protocol.

### 2.7 16S SSU rRNA gene amplicon sequencing

#### 2.7.1 First stage PCR amplification

16S SSU rRNA genes were amplified using two sets of primers, targeting either bacterial V3–V4 (HF samples: S‐D‐Bact‐0341‐b‐S‐17—5′ CCTACGGGNGGCWGCAG 3′ and S‐D‐Bact‐0785‐a‐A‐21—5′ GACTACHVGGGTATCTAATCC3′) or bacterial and archaeal V4 (THW samples: S‐D‐Arch‐0519‐a‐S‐15—5′ CAGCMGCCGCGGTAA 3′ and S‐D‐Bact‐0785‐b‐A‐18—5′ TACNVGGGTATCTAATCC 3′) regions of 16S rRNA genes (Klindworth et al., 2013). The 50 µl amplification reaction for HF samples consisted of 1 µl of each forward and reverse primer (10 µM), 5 µl of 10× reaction buffer, 1 µl of 10 mM dNTP mixture and 0.25 µl Taq polymerase (New England Biolabs, 5,000 U/ml) and DNA template (from soil, spread and iChip at various concentrations). PCR reaction conditions for HF samples were as follows: initial denaturation at 95°C for 5 min, 30 cycles consisting of denaturation at 95°C for 30 s, annealing at 55°C for 30 s and extension at 68°C for 60 s and final extension at 68°C for 5 min. Samples from the THW site were amplified with 0.5 µl Q5 High‐Fidelity DNA polymerase (New England Biolabs, 2,000 U/ml) in the presence of DNA template (from soil, spread or iChip plates at various concentrations), 1 µl of each forward and reverse primer (10 µM), 10 µl of 5× reaction buffer and 1 µl of 10 mM dNTP mixture in 50 µl reaction. Reactions were initiated by denaturation at 98°C for 2 min, followed by 30 cycles of denaturation (98°C, 5 s), annealing (50°C, 30 s) and extension (72°C, 30 s), terminated by final extension at 72°C for 5 min. The size of the PCR products (HF: *c*. 450 bp, THW: 270 bp) was confirmed by agarose gel electrophoresis. All PCR products were purified using 0.8 volumes of Agencourt AMPure XP magnetic beads (Beckman Coulter), washed twice with freshly made 80% (v/v) ethanol and eluted with 40 µl of DNase/RNase‐free water. Clean PCR products were quantified using a dsDNA high sensitivity assay kit for Qubit fluorometric system.

#### 2.7.2 Library preparation

Illumina libraries were prepared using a Nextera XT kit, following the manufacturer's recommendations for 16S rRNA gene PCR amplicon barcoding (using 2× NEBNext High‐Fidelity PCR master mix), clean up and pooling. Indexed libraries were quantified using Qubit, diluted to 4 nM with EB buffer (10 mM Tris‐Cl, pH 8.5) and barcoded samples were mixed in equal volumes. Pooled libraries and PhiX control were denatured with freshly made 0.2 N NaOH, diluted to 4 pM with hybridization buffer and mixed in a 4:1 ratio. The sample was heated (96°C, 2 min), cooled (5 min, 4°C), then immediately loaded on a MiSeq v2 cartridge for 2 × 250 bp paired‐end sequencing.

#### 2.7.3 Data analysis

Demultiplexed, FastQ files were filtered by removing poor quality (*Q* < 25) and short reads (min_HF_ = 400 bp, min_THW_ = 270 bp) using the FastX Toolkit (fastx_clipper command). To ensure comparable data for analysis, the reads for the HF site covering the V3–V4 region of 16S rRNA were converted into reverse‐complement counterparts (fastx_reverse_complement), trimmed to 270 bp to match the V4 amplicons and converted back to complement strands. All combined reads were clustered into operational taxonomic units (OTUs) at 97% shared identity using the GreenGenes database (13_8) as a reference and uclust method (Edgar, 2010) in QIIME (pick_open_reference.py). OTUs with <10 reads were removed from the biom table. Rarefaction curves, alpha diversity metrics and UniFrac distances (Lozupone & Knight, 2005) calculated using QIIME (Caporaso et al., 2010) employed re‐sampling at the level of 19,000 reads—the size of the smallest library—to avoid sample size‐based artifacts (Lozupone, Lladser, Knights, Stombaugh, & Knight, 2011).

To aid analysis, 60,000 16S rRNA gene sequences were subsampled from the dataset of each treatment for classification using SSuMMo (Leach, Chong, & Redeker, 2012). We selected species that were at least 0.2% (120 reads) of the analysed datasets and compared these data using comparative_results.py (part of the SSuMMo package). The headers of the resulting files were edited and then uploaded to iTOL (https://itol.embl.de; Letunic & Bork, 2016) for visualization.

## 3 RESULTS

### 3.1 Diversity and richness of soil, spread plate and iChip‐recovered microbial communities examined by 16S rRNA amplicon sequencing

A total of 20 iChips were used for isolation of microbes from the HF and THW sites, with a potential yield of 7,720 colonies. In most cases only 30%–40% of the wells were filled with colonized agar plugs, which were subsequently transferred onto soil‐extract agar plates. An average of 43% of the plated plugs showed growth after 2 weeks’ incubation at room temperature.

**Figure 2:**
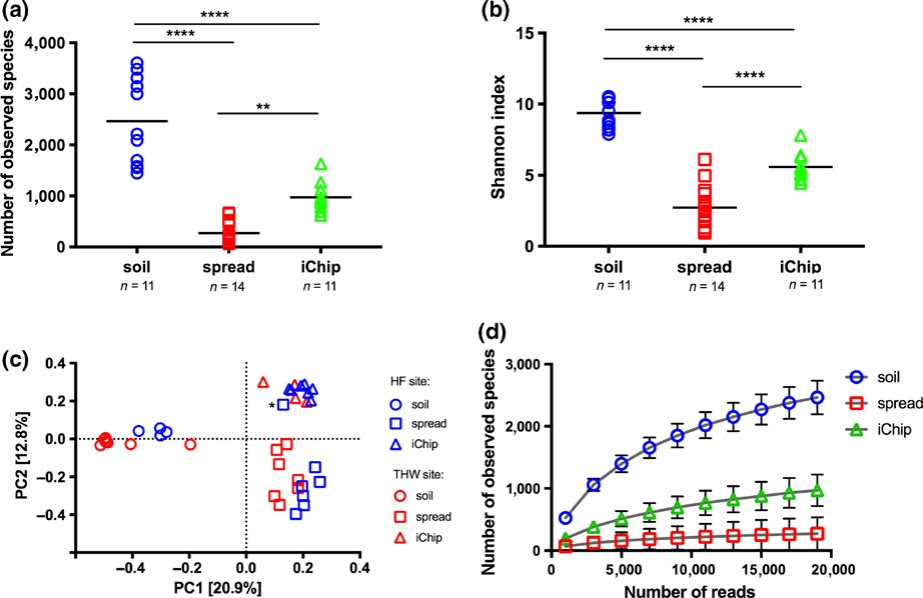
Bacterial community analysis of soil samples and colonies recovered using spread and iChip isolation techniques. The number of observed species (a) and Shannon diversity index (b) were used to determine the richness and diversity of bacterial communities in soil, grown on spread plates and recovered via iChip cultivation. An ordinary one‐way ANOVA was performed for (a) and (b) with Tukey's multiple comparison test with **p = 0.0051, ****p < 0.0001 as indicated. Horizontal lines on the graphs represent mean values. Principal coordinates analysis (c) of unweighted UniFrac indices at the operational taxonomic unit (OTU) level was used to visualize grouping patterns between sequenced samples from the Hagg Farm (HF) and Three Hagges Wood Meadow (THW) sites. The asterisk indicates a nominally spread plate sample that clustered closely with the iChip samples. Rarefaction analysis (d) was performed to estimate species richness based on the number of OTUs for a given sequencing depth (min = 1,000, max = 19,000 reads). Error bars indicate *SEM* (*n*_soil_ = 11, *n*_spread_ = 14, *n*_iChip_ = 11)

16S rRNA gene amplicon sequencing was used to characterize the microbial populations present in soil samples, colonies recovered using traditional spread plates, and in situ iChip methods. Thirty‐nine libraries (three libraries per student) were prepared and sequenced using the Illumina MiSeq platform (Supporting Information Table S1), resulting in 4.8 million paired‐end reads with a mean 124,567 reads per sample (*n* = 39, *SEM* = 11,316). Of the 39 libraries obtained, three soil‐derived libraries either showed a low number of reads with poor‐quality *Q* scores or failed paired‐end merging, and consequently were removed prior to further analysis. After quality filtering, 3.2 million high‐quality reads (mean = 89,285 reads, *SEM* = 8,183) were used for OTU picking and taxonomic classification. The OTU table was subsampled to 19,000 reads to demonstrate that the sequencing effort was sufficient to give an accurate estimate of microbial diversity. The number of observed species for iChip samples was 3.6‐fold higher than for traditional spread plate cultivation (*p* < 0.001, Figure 2a, Table 1). Soil samples showed the greatest number of observed species with an average of 2,465 OTUs (*n* = 11, *SEM* = 256). The iChip method enabled recovery of a greater proportion of the species originally found in the soil samples (39%, *n* = 11, mean = 970 OTUs, *SEM* = 85) compared to traditional spread plating (11%, *n* = 14, mean = 272 OTUs, *SEM* = 56). Similarly, Shannon diversity index (Table 1) demonstrated greatest diversity in soil samples and lowest in traditionally cultured samples (Figure 2b). Principal coordinate analysis was used to compare the identified community members between soil, spread and iChip samples (Figure 2c). Based on PC1, the soil communities contain significantly different microorganisms than the communities retrieved using culturing methods. Similarly, PC2 separated communities from spread and iChip culturing methods into two distinct clusters (Figure 2c). Rarefaction analysis indicated that the sequencing effort in our study was sufficient to provide accurate estimate of bacterial diversity across all samples (Figure 2d).

**Table 1:** Richness and diversity of soil, spread plate and iChip recovered microbial communities from HF and THW sites

Site	HF			THW		
Sample	*n*	Observed species	Shannon index	*n*	Observed species	Shannon index
Soil	4	1,733 (±167.3)	8.3 (±0.16)	7	2,883 (±290.5)	10 (±0.23)
Spread	7	237 (±70.7)	2.2 (±0.37)	7	308 (±91.2)	3.3 (±0.7)
iChip	7	846 (±63)	5.1 (±0.16)	4	1,183 (±172.2)	6.4 (±0.5)

Notes

HF: Hagg Farm; THW: Three Hagges Wood Meadow.

*n*: number of samples, in brackets *SEM*.

### 3.2 Taxonomic evaluation of recovered microbial communities

**Figure 3:**
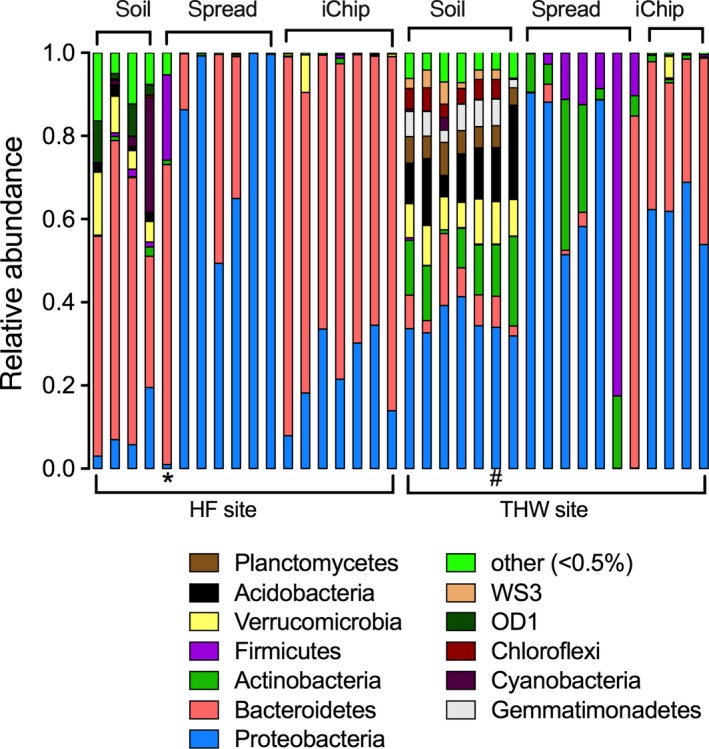
Taxonomic phylum distribution of bacterial communities in soil samples and colonies recovered using spread and iChip isolation techniques from Hagg Farm (HF) and Three Hagges Wood Meadow (THW) sites using 16S rRNA gene amplicon sequencing. The spread plate sample marked with * showed more similarity to the iChip samples based on phylum distribution and PCoA (Figure 2c). The soil sample marked with # appears to have been sequenced twice

To examine the taxonomic structure of bacterial communities in our samples, a taxonomic classification was performed using the GreenGenes (gg_13_5) database (McDonald et al., 2012). This enabled identification of 42 bacterial phyla, among which 12 phyla showed average relative abundance across all the samples higher than 0.5%. Proteobacteria and Bacteroidetes were the dominant phyla in soil communities (Figure 3). These results provided a further opportunity to examine how the bacterial soil communities varied between sites and PCR amplification strategy since two different sets of primers were used in this work. The proportion of Proteobacteria and Bacteroidetes phyla in soil samples was significantly different between HF and THW sites, with a higher abundance of Proteobacteria at the THW site (THW: 35.3% vs. HF: 8.8%, *p* < 0.0001) and Bacteroidetes at the agricultural HF site (THW: 7.5% vs. HF: 55.1%, *p* < 0.0001). The relative abundance of these phyla showed a similar distribution in iChip‐recovered communities; enrichment of Bacteroidetes members for HF (HF: 75% vs. THW: 35.2%, *p* < 0.001) and Proteobacteria assigned OTUs for THW site was observed (THW: 61.7% vs. HF: 22.8%, *p* < 0.001)*.* Overall, Proteobacteria dominated most of the samples derived from spread plates in both examined sites. In addition, a high abundance of Actinobacteria (THW:14.4% vs. HF: 0.3%) and Firmicutes (THW:18.2% vs. HF: 3%) in THW‐derived spread plates was noted compared to a limited abundance in HF‐derived spread plates samples.

**Figure 4:**
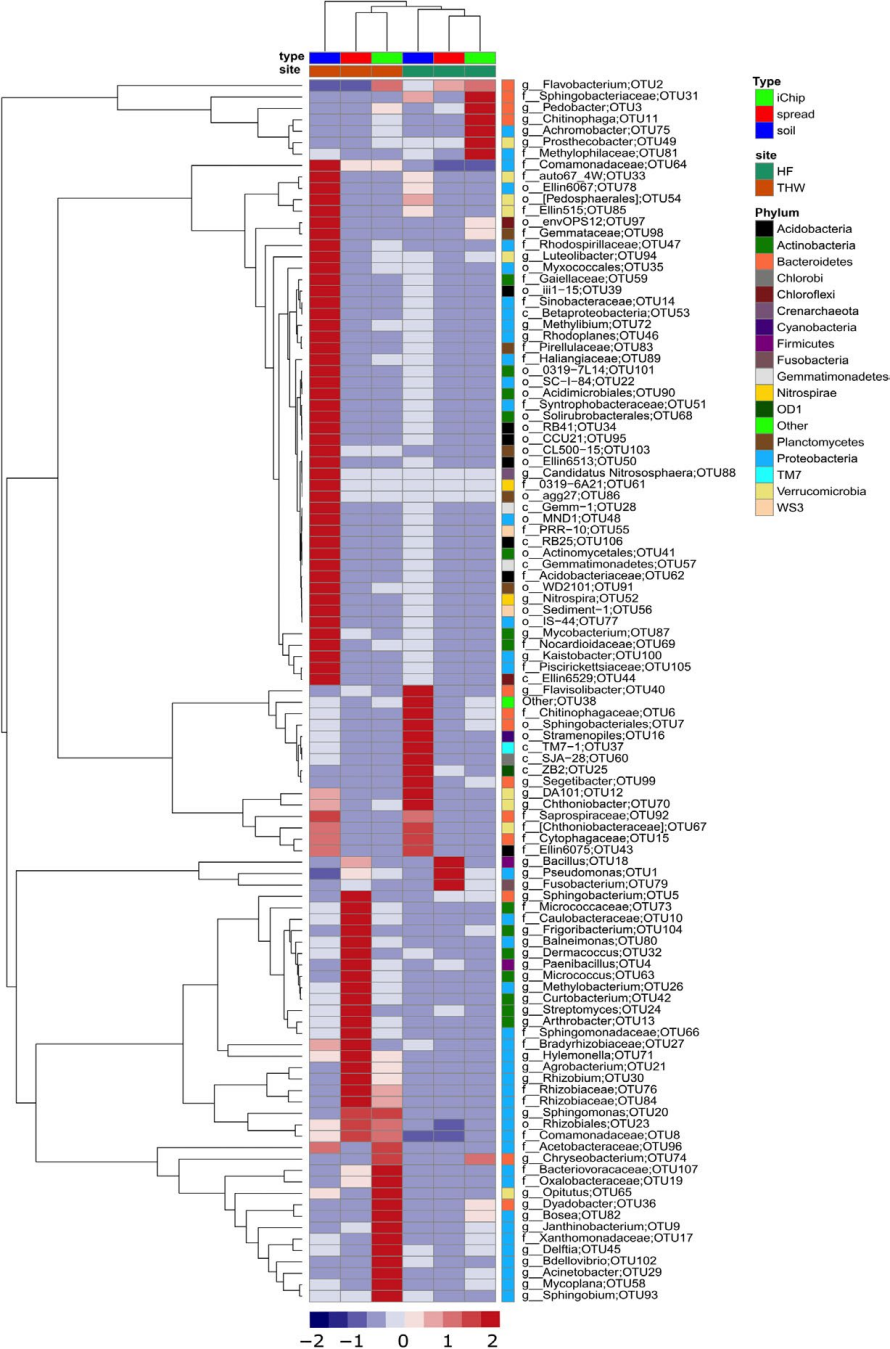
A heatmap of bacterial genera in soil, spread and iChip retrieved microbial communities based on 16S rRNA amplicon sequencing. Columns with similar annotations were collapsed by calculating the mean for each group. Rows depict identified operational taxonomic units (OTUs) with a summed relative abundance >0.1%. Row names represent the lowest taxonomic rank for a given OTU: g—genus, f—family, o—order, c—class. Rows were centered by subtracting the row means (omitting NAs) of OTUs from their corresponding row; scaling was performed by dividing the (centered) row of OTUs by their standard deviations. The relative abundance of an OTU to which unit variance scaling was applied, in soil, spread and iChip recovered microbial communities ranges from −2 to 2 as shown in the lower heatmap key. Rows were clustered using Euclidean distance and average linkage. Columns were clustered using correlation distance and average linkage. The heatmap was constructed using R pheatmap package (Metsalu & Vilo, 2015).

The taxonomic hierarchy across all sampled soil, spread and iChip‐derived bacterial communities was examined at genus level returning 665 genera. The average relative abundance of 107 genera was higher than 0.1% based on all samples and these were further analysed (Figure 4). The abundance of these genera accounted for >90% of relative abundance for all samples examined apart from the THW soil samples where they accounted for 74%. Out of 107 genera, HF and THW soils shared 99 but their abundances were differently distributed. Soil from the HF site was dominated by OTUs assigned to Chitinophagaceae (*n* = 4, 18.1% *SEM* = 2.1) and Sphingobacteriales (*n* = 4, 16.8% *SEM* = 5.9). OTUs assigned to family Sinobacteraceae (*n* = 7, 4.2%, *SEM* = 1) and order SC‐I‐84 of the beta‐proteobacteria (*n* = 7, 3.2%, *SEM* = 0.5) dominated the THW soils. The abundance of Chitinophagaceae was significantly higher at the HF site than the THW site (*n* = 7, 2.2%, *p* < 0.001). Other groups, which showed significant difference between both sites, were OTUs assigned to *Flavobacterium* (HF: 10.9% vs. THW: 0.5%), *Stramenopile* (HF: 7.5% vs. THW: 0.1%) and class ZB2 of OD1 phylum (HF: 5.1% vs. THW: 0%). *Pseudomonas* was the most abundant genus recovered from both sites through spread plate cultivation (*n* = 7, HF: 70.5% vs. THW: 22.6%, *p* < 0.0001). *Flavobacterium* species also showed a high abundance in the HF‐derived spread plates in contrast to the THW‐derived spread plates (*n* = 7, HF: 18% vs. THW: 0.02%, *p* < 0.0001). The THW‐derived spread plates also yielded OTUs assigned to *Paenibacillus* (15.8%), *Sphingobacterium* (12.1%) and Caulobacteraceae (7.2%). The taxonomic profile for the most abundant bacterial species recovered using iChips was different from traditional spread plate culturing. HF‐ and THW‐derived iChip plates were dominated by *Pedobacter *(HF: 39.9% vs. THW: 9.3%), *Flavobacterium* (HF: 22.4% vs. THW: 20.8%) and *Pseudomonas* (HF: 16.4% vs. THW: 17.3%).

To confirm that iChip cultivation was superior to traditional spread plate culturing, we compared the OTUs present in 85% of our samples (e.g. six out of seven iChip samples must contain a specific OTU to be retained for analysis). For HF‐derived samples, the majority of OTUs (81.3%) were unique to iChip plates and not identified using spread plates. For THW‐derived samples, spread plating failed to culture any unique OTUs and the majority of OTUs were identified using iChip isolation.

**Figure 5:**
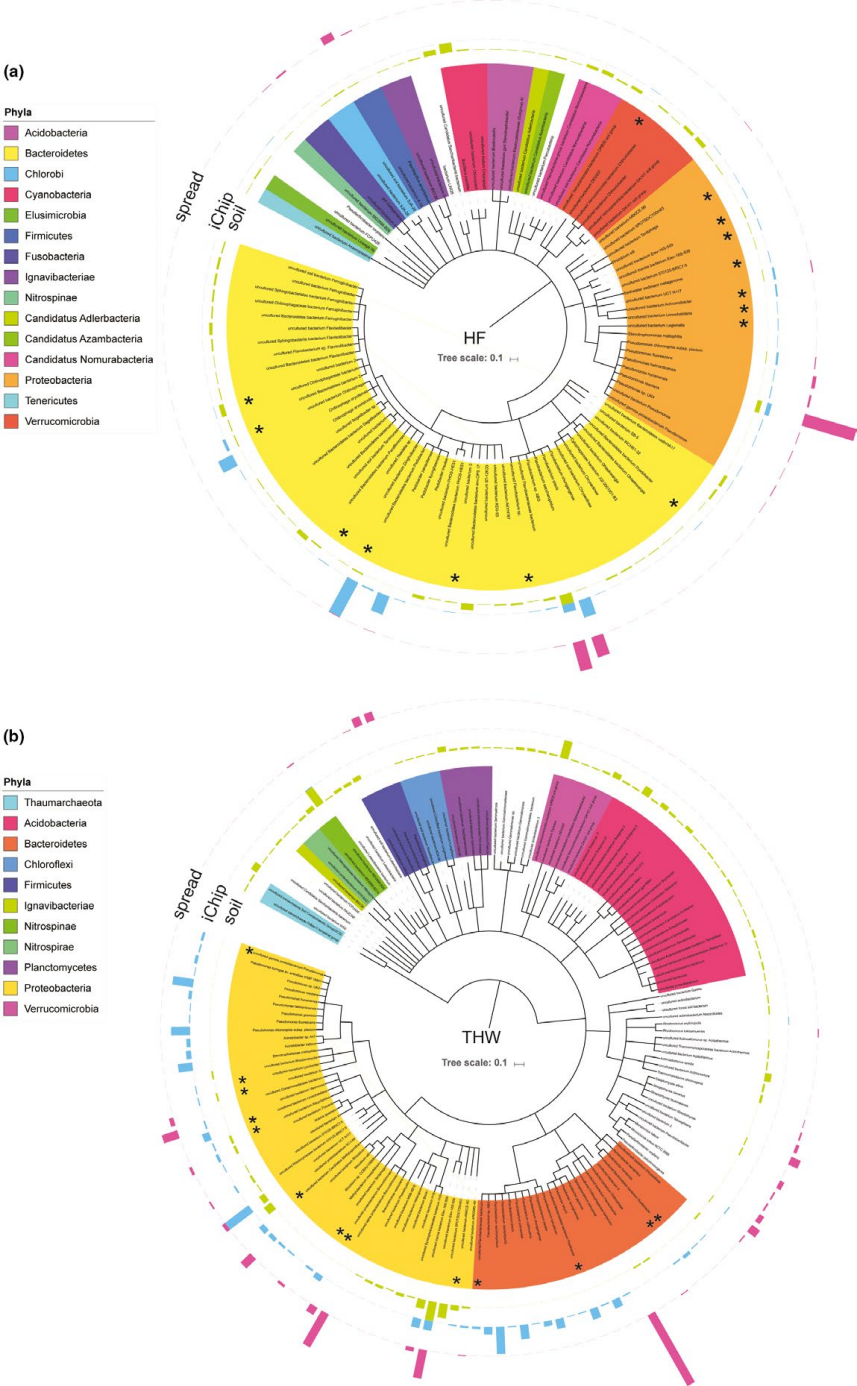
Phylogeny of species based on SSuMMo analysis of 60,000 16S rRNA gene sequences sampled from the collected datasets. Only organisms that were at least 0.2% of the analysed reads are included in the trees. Species previously annotated as “uncultured” are indicated with an asterisk (*). Bar heights indicate the relative abundance of reads within each sample. (a) Hagg Farm (HF) samples, (b) Three Hagges Wood Meadow (THW) samples. Species names are provided in Supporting Information Table S3

We further examined the recovered 16S rRNA gene sequences using SSuMMo (Leach et al., 2012), which classifies sequences from unknown organisms based on their closest known relatives using hidden Markov models. We examined a subset of 60,000 sequences per treatment and directly compared the abundance of the organisms identified in this way. Using a threshold of at least 120 matching reads (0.2% abundance) to simplify visualization, we generated phylogenetic trees using iTOL (Letunic & Bork, 2016) (Figure 5). Our results confirmed that the use of iChips allowed the individual cultivation of species previously reported as uncultured, regardless of their abundance in the original soil sample. As observed in our QIIME analysis, these uncultured organisms were mainly from the phyla *Proteobacteria* and *Bacteroidetes.* Of the abundant species grown in iChips, uncultured species represented between 8.5% (THW, Figure 5b) and 14.5% (HF, Figure 5a). Five of the uncultured species grown in iChips (*Tardiphaga*, *Limnohabitans*, *Dyadobacter*, *Pedobacter* and “bacterium 3”) were isolated in this manner from both experiments although they were not detected on spread plates.

### 3.3 Screening for antimicrobial activities

Based on the increased diversity of species that grew on conventional media following iChip incubation, colonies were replica plated and overlaid with indicator species so that these organisms could be screened for antimicrobial metabolites via the production of clearing zones. In total, 56 colonies were identified by students as active against at least one of the ESKAPE indicators. These were streak purified and rescreened to confirm their antimicrobial potential. Two isolates consistently showed antimicrobial activities. Based on 16S rRNA gene sequencing, isolate CFO_SW1(3) was related to *Bacillus subtilis* strain kp6 (MH200633.1) and displayed inhibitory activity against *E. coli.* Isolate RH6B(8c) showed high similarity to *Delftia* sp. (FR682925.1) and generated clearing zones indicative of antimicrobial activity against *E. coli*, *P. putida* and *E. aerogenes *(Table 2). Additional characterization of these isolates was beyond the scope of this work.

**Table 2:** Antimicrobial activities for two iChip‐recovered isolates CFO_SW1(3) and RH6B(8c) tested against ESKAPE indicators

ESKAPE indicator	CFO_SW1 (3)	RH6B(8c)
*Escherichia coli *ATCC 11775	✓ 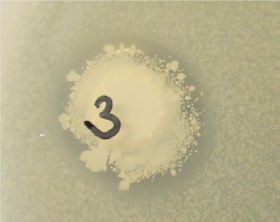	✓ 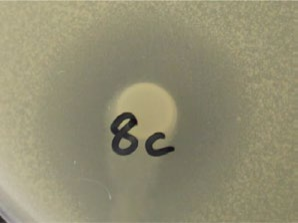
*Pseudomonas putida *ATCC 12633	n.d.	✓ 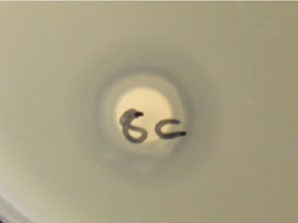
*Enterobacter aerogenes *ATCC 51697	n.d.	✓ 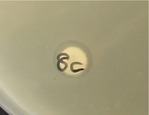
*Enterococcus raffinosus *ATCC 49464	n.d.	n.d.
*Staphylococcus epidermidis *ATCC 14990	n.d.	n.d.

Note

n.d.: not detected.

## 4 DISCUSSION

Here we report the development of a practical that seeks to provide research‐based molecular ecology experience to undergraduates while introducing them to two challenging microbiological issues: the great plate count anomaly and a need to identify new antibiotics. Two cohorts of undergraduate students in the third year of a 4 year taught Masters course worked in groups over 18 weeks to isolate microorganisms with potentially novel antimicrobial properties from soil through the application of traditional and novel microbiological techniques. The evaluation of microbial identities was performed using high throughput 16S rRNA gene amplicon sequencing, resulting in large datasets for the students to analyze and interpret.

### 4.1 iChips facilitate the recovery of antimicrobial producers

Visual observation that the number of colonies recovered from iChips were higher than those observed on spread plates corresponded well with the alpha‐diversity measurements calculated from sequencing data. Both cohorts independently concluded that a higher number of more diverse bacterial species was recovered using the iChip isolation method compared to traditional spread plating techniques. As previously reported, the in‐situ cultivation method offered by iChips facilitates the culturing of a greater diversity of microorganisms from various environments compared to traditional methods (Nichols et al., 2010). In this study, the iChip strategy led to the isolation of two microorganisms with confirmed antimicrobial activities. Isolate CFO_SW1(3) was related to *B. subtilis*—a low G + C, Gram‐positive *Firmicutes* that has been commonly used for decades as a model microorganism for genetic and biochemical studies of chromosome replication and bacterial sporulation (Kunst et al., 1997). The *Bacillus* genus produces a wide assortment of biologically active small molecules with a range of antagonistic activities, including antibacterial non‐ribosomal cyclic lipopeptides of the surfactin and gageotetrin families, polyketides such as macrolactin and bacillaene, antitumor polyketide‐peptide hybrids like amicoumacin and ieodoglucomide, and the discoipyrrole alkaloids (Abriouel, Franz, Omar, & Gálvez, 2011; Stein, 2005). *Bacillus* species are routinely isolated from soil (Yilmaz, Soran, & Beyatli, 2006) but are also associated with decaying organic material such as compost, manure and hay (Earl, Losick, & Kolter, 2008).

The second isolate, RH6B(8c), was related to *Delftia* sp. which have been the subject of only limited studies as a potential producer of antimicrobial agents. Gene loci that might encode for resorcinol, terpenes, and a bacteriocin (all with potential antimicrobial properties) were found in the genome of *Delftia acidovorans* RAY209 (Perry et al., 2017) and *Delftia tsuruhatensis* MTQ3 (Hou et al., 2015). *Delftia* species are Gram‐negative, aerobic, rod‐shaped, motile bacteria within the order Burkholderiales of the class Betaproteobacteria. *Delftia* isolates have been reported as accumulators of poly‐B‐hydroxybutyrate—a carbon and energy storage material used during depletion of the exogenous carbon sources, that can serve as a cryoprotectant of bacterial cells in low temperature conditions and provides protection against oxidative stress (Obruca, Sedlacek, Koller, Kucera, & Pernicova, 2017). A wide range of enzymatic activities including peptidoglycan‐degrading enzymes (Jørgensen, Brandt, Nybroe, & Hansen, 2009) have been identified within this genus and clearly its biotechnological potential should be further explored (Morel, Iriarte, Jara, Musto, & Castro‐Sowinski, 2016).

### 4.2 Detecting representative diversity

The taxonomic evaluation of soil, spread and iChip recovered microbial communities highlighted the biases associated with our amplicon sequencing methodologies. Since our two cohorts of students sampled different experimental sites (HF vs. THW) and used different primer sets and polymerases to either amplify the V3–V4 or the V4 regions of the 16S rRNA genes, a direct comparison of the microbial community profiles we recovered was not possible. However, it was noted that cohort one (HF site) consistently reported a high abundance of Bacteroidetes in soil samples (Figure 3) compared to cohort two (THW site). Based on previous reports (Fierer, 2017), the soil microbiome is dominated by taxa affiliated with Acidobacteria, Verrucomicrobia and Proteobacteria, with Bacteroidetes accounting for approximately 10% of the soil microbiome. Several factors might have resulted in the disproportionately high numbers of Bacteroidetes in the soil community structure of the HF samples compared to published reports. Primer bias is known to cause over‐ and/or under‐representation of certain taxa in amplicon sequencing results (Sun, Jiang, Wu, & Zhou, 2013; Thijs et al., 2017). Thus, the V3–V4 primers used to amplify DNA from the HF site could have resulted in the overestimation of Bacteroidetes in our HF soil samples. Another possibility might be contaminating DNA from the extraction kit used to analyze the HF samples (Salter et al., 2014). Based on these observations, in our second iteration of this practical the THW cohort performed 16S rRNA gene amplification with primers targeting the V4 region. This approach resulted in similar soil‐community profiles to other soil‐microbiome studies (Lanzén et al., 2016; Thompson et al., 2017; Tian et al., 2017).

By separately analyzing a subsample of the aggregated data collected by both cohorts of students using SSuMMo to assign 16S rRNA gene sequences to their closest species (Leach et al., 2012) and considering only relatively abundant organisms (at least 0.2% of analysed sequences) to simplify visualization, we demonstrated that the iChip approach facilitated the effective culturing of at least 28 species previously described as “uncultured”, five of which were isolated consistently from both iterations of the experiment. iChips facilitated the growth of a different range of species to traditional plating methods, potentially providing access to new antimicrobial molecules as previously reported (Ling et al., 2015). Analysis by SSuMMo suggested that the “uncultured” species grown in iChips and consequently subcultured on solid media were skewed toward Bacteroidetes and Proteobacteria. Modifications to the solid media composition, method of media preparation, or length of incubation could all influence these outcomes. For example, it has been recently reported that media autoclaved in the presence of phosphate (inevitably present in the soil agar we used here) reduces the growth of organisms susceptible to oxidative stress (Kato et al., 2018).

### 4.3 Protocol pitfalls and improvements

Our first experiments indicated a slightly atypical distribution of soil species. In addition, contamination of our negative controls (where iChips were loaded only with agarose) was noted for the HF samples and was attributed to carrying out the assembly of these controls at the field site, rather than under sterile laboratory conditions. Based on these observations, in our second iteration of this practical the THW cohort performed 16S rRNA gene amplification with primers targeted to the V4 region and used the higher fidelity Q5 polymerase. This approach resulted in similar soil‐community profiles to other soil‐microbiome studies (Lanzén et al., 2016; Thompson et al., 2017; Tian et al., 2017). We maintained sterility in these negative controls by returning soil samples to the laboratory and assembling the iChips in laminar flow cabinets before returning the samples to the field for initial growth.

Other interpretational challenges were likely due to human error: mistakes in sample labelling were difficult to confirm definitively, but were supported by the obvious differences in relative species abundance between samples in different categories in one case (Figure 3, sample marked with *, where an iChip sample appears to have been labelled as a spread sample) and the unexpected similarity between samples in another (Figure 3, sample marked with #, where a soil sample appears to have been sequenced twice).

Additional improvements could be made to the methodology we describe here: students found the overlay method technically challenging and would benefit from additional practice on non‐critical samples to master this technique. We used an approximation for the number of cells in our soil samples based on a series of separate observations. This could be improved through the accurate quantification of the specific soil samples used. Cell counts could be obtained via DNA staining of cells using a THOMA counting chamber as described above or through microbial flow cytometry if these facilities are readily available (Frossard, Hammes, & Gessner, 2016). As previously reported (Davis et al., 2017) students could probe the diversity of culturable organisms by plating soil and iChip contents onto specialized media to target, for example the growth of known antibiotic producers such as *Streptomycetes*. They could also consider the separate preparation of phosphate for addition to media and the use of sterile rainwater rather than PBS for soil dilutions.

### 4.4 Costs and effectiveness

We estimate the total cost of these investigations at approximately £250 per student for a practical that demanded effort of 1–2 days per week for 18 weeks. These costs do not include the initial outlay for fabrication of the reusable iChip devices, or travel to field sites, both of which are variable and relatively negligible costs (Supporting Information Table S2). These costs could be further reduced by increasing the number of students/samples sequenced per run (sufficient sequences could still be obtained) and by having students work in pairs.

Together, the experiments and associated analyses introduced students to the use of iChips, provided practical experience of DNA extraction methodologies, PCR, high throughput sequencing and exposure to bioinformatics tools for microbial community analyses. All 13 of the students who carried out these protocols successfully recovered and amplified metagenomic DNA from at least a subset of the samples they collected. They gained a better appreciation of field and lab work as well as benefitting from directly manipulating and visualizing their own data. Their results provided them with practical, real‐world illustrations of rarefaction curves, alpha‐ and beta‐ species diversity, Shannon diversity indices and, the concepts of species richness and evenness. These were then communicated in a written report, allowing both staff and students to assess the effectiveness of this exercise. The resulting reports indicated that students had understood the ecological and molecular concepts well and were able to communicate and interpret their results effectively. Overall, we consider this as a cost‐effective method of supporting the teaching of the relevant practical and analytical skills.

## CONFLICT OF INTEREST

None declared.

## AUTHORS CONTRIBUTION

AMA, KRR and JPJC designed the work and assisted in the acquisition of the data. AMA and JPJC analysed the data. AMA and JPJC drafted the manuscript, AMA, KRR and JPJC revised and approved the work.

## DATA ACCESSIBILITY

Raw reads from 16S amplicon sequencing deposited to European Nucleotide Archive (ENA) and available under accession number PRJEB26611.

Sampling locations and other metadata are available from Dryad, https://doi.org/10.5061/dryad.cq4pv11.
