# Supplementary material for: A practical introduction to microbial molecular ecology through the use of isolation chips
Source: Ecol Evol. 2018 Dec 11;8(24):12286–98. doi: 10.1002/ece3.4748 (PMC6309002; doi:10.1002/ece3.4748)
Supplement: Supplementary file 1 [file ECE3-8-12286-s001.docx]

**Appendix**

**Supplementary Figure 1.** Maps of the field sites used during the study. Hagg Farm (HF, Askham Bryan, 53°56’16’’N, 1°10’10’’W) and Three Hagges Wood Meadow (THW, 53°50’51’’N, 1°2’50’’W) are marked in orange polygon. Points on the map indicate an approximate location of iChip burials.

**Supplementary Figure 2.** Isolation Chip used in the study and colonies recovered from iChip grown on soil-agar plates.

Isolation Chip consists of three parts: top, middle and bottom that are stacked up and assembled. Top and bottom of the middle part is covered with a membrane that enables diffusion of nutrients that support growth of individual isolate in the well (1 μl).

**Supplementary Table 1**. Number of 16S SSU rRNA gene reads generated from MiSeq run during the study.

Samples marked with * were excluded from the analysis due to low number or poor quality of reads. All the samples were submitted to the European Nucleotide Archive (ENA) and are available under accession number PRJEB26611.

|  | Number of reads | | |  |  |
| --- | --- | --- | --- | --- | --- |
| ID | Raw | Merged | Filtered | Description | Site |
| CF1 | 230931 | 177226 | 119548 | soil | HF |
| CF2 | 181988 | 129525 | 81299 | spread | HF |
| CF3 | 153148 | 121454 | 95604 | iChip | HF |
| CL1 | 184767 | 122754 | 95173 | soil | HF |
| CL2 | 140707 | 127522 | 99721 | spread | HF |
| CL3 | 156770 | 113590 | 98939 | iChip | HF |
| CO1* | 25950 | 8831 | 3060 | soil | HF |
| CO2 | 78917 | 56220 | 46411 | spread | HF |
| CO3 | 157251 | 136556 | 122388 | iChip | HF |
| GA1 | 105295 | 47498 | 36348 | soil | HF |
| GA2 | 198166 | 186778 | 178263 | spread | HF |
| GA3 | 188162 | 151512 | 119096 | iChip | HF |
| L1* | 55362 | 29131 | 15297 | soil | HF |
| L2 | 184866 | 118777 | 47618 | spread | HF |
| L3 | 180929 | 109179 | 66213 | iChip | HF |
| M1* | 104844 | 43016 | 14397 | soil | HF |
| M2 | 385412 | 303840 | 265058 | spread | HF |
| M3 | 212270 | 164191 | 140832 | iChip | HF |
| Z1 | 113634 | 50986 | 34908 | soil | HF |
| Z2 | 99822 | 58627 | 43302 | spread | HF |
| Z3 | 146327 | 87186 | 63874 | iChip | HF |
| B1 | 56386 | 56158 | 50243 | soil | THW |
| B2 | 66722 | 66457 | 63264 | spread | THW |
| B3 | 57971 | 57754 | 54812 | iChip | THW |
| E1 | 79000 | 78623 | 71590 | soil | THW |
| E2 | 66562 | 66215 | 63325 | spread | THW |
| E3 | 63720 | 63458 | 60599 | iChip | THW |
| H1 | 114061 | 113593 | 103808 | soil | THW |
| H2 | 168378 | 167647 | 158076 | spread | THW |
| H3 | 66024 | 65761 | 62033 | iChip | THW |
| R1 | 49170 | 48952 | 43531 | soil | THW |
| R2 | 47770 | 47601 | 45172 | spread | THW |
| R3 | 88604 | 88124 | 83854 | iChip | THW |
| V1 | 86283 | 85775 | 77746 | soil | THW |
| V2 | 59357 | 59125 | 56297 | spread | THW |
| V3 | 196275 | 195473 | 173684 | soil | THW |
| VC1 | 161647 | 160981 | 148813 | soil | THW |
| VC2 | 62609 | 62301 | 58790 | spread | THW |
| VC3 | 82062 | 81699 | 77885 | spread | THW |
| Sum | 4,858,119 | 3,910,096 | 3,240,871 |  |  |
| Mean | 124,567.1 | 100,258.9 | 83,099.2 |  |  |

**Supplementary Table 2. Breakdown cost of the consumables for iChip project.**

The materials required for the experimental part of the project are listed. Total price is based on supplier’s price in April 2018. Catalogue numbers for selected consumables are indicated by #. Price per student, was calculated based on the maximum number of samples/reactions prepared by a student during the project. Estimated overall expenditure per student is c.a. £260.

| **Consumables** | **Total price [£]** | **Price per student [£]** | **Equivalent per student** |
| --- | --- | --- | --- |
| Whatman™ 47mm Nuclepore™ Polycarbonate Track-Etched Membranes membranes #111103 (100) | 150 | 15 | 10 membranes |
| iChip design and manufacture (1) | 20 |  |  |
| ESKAPE (5 strains) | 425 |  |  |
| Qiagen PowerSoil Dneasy kit # 12888-100 (100 samples) | 416 | 41.6 | 10 samples |
| Qiagen UltraMicrobial Dnaeasy kit # 12224-50 (50 samples) | 118 | 23.6 | 10 samples |
| Primers | 10 |  |  |
| NEB Q5 polymerase # M0491S (100 U) | 76 | 7.6 | 10 reactions |
| Agencourt AMPure XP beads #A86630 (5 ml) | 200 | 20 | 500 ml |
| NEBNext PCR master mix #M0541S (50) | 90 | 9 | 5 reactions |
| Illumina Nextera XT #FC-131-1002 (96 indexes or 384 samples) | 770 | 6 | 3 samples |
| Illumina MiSeq kit V3 2x300 (600-cycle) #MS-102-3003 | 1220 | 40 | 3 samples |
| General consumable (plates, rods,tubes, tips, media etc) |  | 100 |  |
| Total | **3475** | **262.8** |  |

**Supplementary Table 3. Species labels for Figure 5 (labelled clockwise from gap).** * indicates uncultured species grown using iChips but not present on spread plates.

| **Sample** | **Phylum** | **Species** |
| --- | --- | --- |
| HF | Tenericutes | uncultured_bacterium_Anaeroplasma |
| HF | *Ca*.Azambacteria | uncultured_bacterium_Lineage_IIb |
| HF |  | uncultured_bacterium_FCPU426 |
| HF |  | Pseudarthrobacter_oxydans |
| HF | Nitrospinae | uncultured_bacterium_MD2898-B26 |
| HF | Fusobacteria | uncultured_bacterium_4 |
| HF | Fusobacteria | gut_metagenome |
| HF | Chlorobi | uncultured_bacterium_SJA-28 |
| HF | Chlorobi | uncultured_soil_bacterium_SJA-28 |
| HF | Firmicutes | uncultured_bacterium_4-15 |
| HF | Firmicutes | Paenibacillus_amylolyticus |
| HF | Ignavibacteriae | uncultured_bacterium_BSV26 |
| HF | Ignavibacteriae | uncultured_bacterium_5 |
| HF |  | bacterium_LWQ8 |
| HF |  | uncultured_Candidatus_Saccharibacteria_bacterium |
| HF | Cyanobacteria | Bacillaria_paxillifer |
| HF | Cyanobacteria | uncultured_bacterium_Chloroplast |
| HF | Cyanobacteria | uncultured_diatom_Chloroplast |
| HF | Acidobacteria | uncultured_bacterium_Blastocatella |
| HF | Acidobacteria | uncultured_bacterium_gp4_Stenotrophobacter |
| HF | Acidobacteria | uncultured_bacterium_Blastocatellaceae_(Subgroup_4) |
| HF | *Ca*.Alderbacteria | uncultured_bacterium_Candidatus_Adlerbacteria |
| HF | *Ca*.Azambacteria | uncultured_bacterium_Candidatus_Azambacteria |
| HF |  | uncultured_bacterium_Parcubacteria |
| HF | *Ca*.Nomurabacteria | uncultured_Parcubacteria_group_bacterium_Candidatus_Nomurabacteria |
| HF | *Ca*.Nomurabacteria | uncultured_bacterium_Candidatus_Nomurabacteria |
| HF | *Ca*.Nomurabacteria | uncultured_soil_bacterium_Candidatus_Nomurabacteria |
| HF | Verrucimicrobia | uncultured_Verrucomicrobia_bacterium_OPB35_soil_group |
| HF | Verrucimicrobia | uncultured_bacterium_DEV007* |
| HF | Verrucimicrobia | uncultured_Verrucomicrobia_bacterium_Chthoniobacter |
| HF | Verrucimicrobia | uncultured_bacterium_Chthoniobacter |
| HF | Verrucimicrobia | uncultured_Verrucomicrobia_bacterium_DA101_soil_group |
| HF | Verrucimicrobia | uncultured_bacterium_DA101_soil_group |
| HF | Proteobacteria | uncultured_bacterium_ARKICE-90 |
| HF | Proteobacteria | uncultured_bacterium_SPOTSOCT00m83 |
| HF | Proteobacteria | uncultured_bacterium_Tardiphaga* |
| HF | Proteobacteria | Rhizobium_etli |
| HF | Proteobacteria | uncultured_bacterium_Elev-16S-509* |
| HF | Proteobacteria | uncultured_marine_bacterium_Elev-16S-509* |
| HF | Proteobacteria | uncultured_bacterium_070125-BRIC7-5 |
| HF | Proteobacteria | freshwater_sediment_metagenome* |
| HF | Proteobacteria | uncultured_bacterium_UCT_N117 |
| HF | Proteobacteria | uncultured_bacterium_Achromobacter* |
| HF | Proteobacteria | uncultured_bacterium_Limnohabitans* |
| HF | Proteobacteria | uncultured_bacterium_Legionella* |
| HF | Proteobacteria | Stenotrophomonas_maltophilia |
| HF | Proteobacteria | Pseudomonas_chlororaphis_subsp._piscium |
| HF | Proteobacteria | Pseudomonas_fluorescens |
| HF | Proteobacteria | Pseudomonas_helmanticensis |
| HF | Proteobacteria | Pseudomonas_hunanensis |
| HF | Proteobacteria | Pseudomonas_reactans |
| HF | Proteobacteria | Pseudomonas_sp._UK4 |
| HF | Proteobacteria | uncultured_bacterium_Pseudomonas |
| HF | Proteobacteria | uncultured_gamma_proteobacterium_Pseudomonas |
| HF | Bacteroidetes | uncultured_bacterium_Bacteroidetes_vadinHA17 |
| HF | Bacteroidetes | uncultured_bacterium_SB-5 |
| HF | Bacteroidetes | uncultured_bacterium_WCHB1-32 |
| HF | Bacteroidetes | uncultured_Bacteroidetes_bacterium_Dyadobacter* |
| HF | Bacteroidetes | uncultured_Bacteroidetes_bacterium_Ohtaekwangia |
| HF | Bacteroidetes | uncultured_bacterium_Ohtaekwangia |
| HF | Bacteroidetes | Cytophagaceae_bacterium_JGI_0001001-B3 |
| HF | Bacteroidetes | uncultured_bacterium_Chryseolinea |
| HF | Bacteroidetes | uncultured_soil_bacterium_Chryseolinea |
| HF | Bacteroidetes | Flavobacterium_chungangense |
| HF | Bacteroidetes | Flavobacterium_piscis |
| HF | Bacteroidetes | Flavobacterium_saccharophilum |
| HF | Bacteroidetes | Flavobacterium_sp._ABG |
| HF | Bacteroidetes | uncultured_Flavobacteriaceae_bacterium |
| HF | Bacteroidetes | uncultured_Flavobacterium_sp.* |
| HF | Bacteroidetes | uncultured_bacterium_AKYH767 |
| HF | Bacteroidetes | uncultured_bacterium_KD3-93 |
| HF | Bacteroidetes | uncultured_bacterium_ST-12K33 |
| HF | Bacteroidetes | uncultured_Bacteroidetes_bacterium_env.OPS_17 |
| HF | Bacteroidetes | uncultured_bacterium_3* |
| HF | Bacteroidetes | uncultured_Bacteroidetes_bacterium_PHOS-HE51 |
| HF | Bacteroidetes | uncultured_bacterium_PHOS-HE51 |
| HF | Bacteroidetes | Pedobacter_insulae |
| HF | Bacteroidetes | Pedobacter_kyungheensis |
| HF | Bacteroidetes | Pedobacter_panaciterrae |
| HF | Bacteroidetes | uncultured_Bacteroidetes_bacterium_Pedobacter* |
| HF | Bacteroidetes | uncultured_bacterium_Dinghuibacter |
| HF | Bacteroidetes | uncultured_Niastella_sp.* |
| HF | Bacteroidetes | uncultured_Bacteroidetes_bacterium_Parafilimonas |
| HF | Bacteroidetes | uncultured_soil_bacterium_Terrimonas |
| HF | Bacteroidetes | uncultured_Bacteroidetes_bacterium |
| HF | Bacteroidetes | uncultured_bacterium |
| HF | Bacteroidetes | uncultured_Bacteroidetes_bacterium_Segetibacter |
| HF | Bacteroidetes | uncultured_Segetibacter_sp. |
| HF | Bacteroidetes | Chitinophaga_arvensicola |
| HF | Bacteroidetes | Chitinophaga_oryziterrae |
| HF | Bacteroidetes | uncultured_bacterium_Chitinophaga* |
| HF | Bacteroidetes | uncultured_Bacteroidetes_bacterium_2 |
| HF | Bacteroidetes | uncultured_Chitinophagaceae_bacterium* |
| HF | Bacteroidetes | uncultured_bacterium_2 |
| HF | Bacteroidetes | uncultured_Bacteroidetes_bacterium_Flavisolibacter |
| HF | Bacteroidetes | uncultured_Flavobacterium_sp._Flavisolibacter |
| HF | Bacteroidetes | uncultured_Sphingobacteriia_bacterium_Flavisolibacter |
| HF | Bacteroidetes | uncultured_bacterium_Flavisolibacter |
| HF | Bacteroidetes | uncultured_Bacteroidetes_bacterium_Ferruginibacter |
| HF | Bacteroidetes | uncultured_Chitinophagaceae_bacterium_Ferruginibacter |
| HF | Bacteroidetes | uncultured_Sphingobacteriales_bacterium_Ferruginibacter |
| HF | Bacteroidetes | uncultured_bacterium_Ferruginibacter |
| HF | Bacteroidetes | uncultured_soil_bacterium_Ferruginibacter |
| THW | Thaumarchaeota | uncultured_crenarchaeote_FHMa11_terrestrial_group |
| THW | Thaumarchaeota | uncultured_crenarchaeote_Soil_Crenarchaeotic_Group(SCG) |
| THW |  | uncultured_bacterium_WS2 |
| THW |  | uncultured_Candidatus_Saccharibacteria_bacterium |
| THW |  | uncultured_bacterium_PAUC34f |
| THW |  | uncultured_bacterium_FCPU426 |
| THW | Ignavibacteriae | uncultured_bacterium_BSV26 |
| THW | Nitrospirae | uncultured_proteobacterium_0319-6A21 |
| THW | Nitrospirae | uncultured_Nitrospiraceae_bacterium_Nitrospira |
| THW | Nitrospinae | uncultured_bacterium_MD2896-B214 |
| THW | Nitrospinae | uncultured_bacterium_MD2898-B26 |
| THW |  | uncultured_Latescibacteria_bacterium |
| THW |  | uncultured_bacterium_Latescibacteria |
| THW |  | uncultured_soil_bacterium_Latescibacteria |
| THW | Firmicutes | uncultured_bacterium_Gelria |
| THW | Firmicutes | uncultured_bacterium_Solibacillus |
| THW | Firmicutes | Paenibacillus_terrigena |
| THW | Firmicutes | Paenibacillus_endophyticus |
| THW | Chloroflexi | uncultured_soil_bacterium_Roseiflexus |
| THW | Chloroflexi | uncultured_bacterium_KD4-96 |
| THW | Chloroflexi | uncultured_Chloroflexi_bacterium_Longilinea |
| THW | Chloroflexi | uncultured_bacterium_Longilinea |
| THW | Planctomycetes | uncultured_bacterium_OM190 |
| THW | Planctomycetes | uncultured_bacterium_Tepidisphaeraceae |
| THW | Planctomycetes | uncultured_bacterium_Gemmata |
| THW | Planctomycetes | uncultured_planctomycete_Pir4_lineage |
| THW | Planctomycetes | uncultured_bacterium_Pirellula |
| THW |  | uncultured_bacterium_Gemmatirosa |
| THW |  | uncultured_bacterium_Gemmatimonadaceae |
| THW |  | uncultured_Gemmatimonas_sp. |
| THW |  | uncultured_bacterium_Gemmatimonas |
| THW |  | uncultured_Gemmatimonadales_bacterium |
| THW |  | uncultured_proteobacterium_2 |
| THW | Verrucomicrobia | uncultured_Verrucomicrobia_bacterium_OPB35_soil_group |
| THW | Verrucomicrobia | uncultured_bacterium_Opitutus |
| THW | Verrucomicrobia | uncultured_bacterium_DEV007 |
| THW | Verrucomicrobia | uncultured_bacterium_Candidatus_Xiphinematobacter |
| THW | Verrucomicrobia | uncultured_Verrucomicrobia_bacterium_DA101_soil_group |
| THW | Verrucomicrobia | uncultured_bacterium_DA101_soil_group |
| THW | Acidobacteria | uncultured_bacterium_Subgroup_17 |
| THW | Acidobacteria | uncultured_bacterium_Subgroup_22 |
| THW | Acidobacteria | uncultured_Acidobacteria_bacterium_Subgroup_2 |
| THW | Acidobacteria | uncultured_Acidobacteriaceae_bacterium_Subgroup_2 |
| THW | Acidobacteria | uncultured_bacterium_Subgroup_6 |
| THW | Acidobacteria | uncultured_proteobacterium_Subgroup_6 |
| THW | Acidobacteria | uncultured_bacterium_Elev-16S-573 |
| THW | Acidobacteria | uncultured_bacterium_RB41 |
| THW | Acidobacteria | uncultured_bacterium_gp4_RB41 |
| THW | Acidobacteria | uncultured_Acidobacteria_bacterium_Bryobacter |
| THW | Acidobacteria | uncultured_bacterium_Candidatus_Solibacter |
| THW | Acidobacteria | uncultured_bacterium_Paludibaculum |
| THW | Acidobacteria | Silvibacterium_bohemicum |
| THW | Acidobacteria | uncultured_bacterium_Candidatus_Koribacter |
| THW | Acidobacteria | uncultured_bacterium_Edaphobacter |
| THW | Acidobacteria | uncultured_bacterium_Granulicella |
| THW | Acidobacteria | uncultured_bacterium_Telmatobacter |
| THW | Acidobacteria | uncultured_Acidobacteriaceae_bacterium_Terriglobus |
| THW | Acidobacteria | uncultured_bacterium_Acidobacteriaceae_(Subgroup_1) |
| THW | Acidobacteria | uncultured_Acidobacteria_bacterium |
| THW | Acidobacteria | uncultured_bacterium |
| THW | Acidobacteria | uncultured_proteobacterium |
| THW |  | uncultured_bacterium_Gaiella |
| THW |  | uncultured_actinobacterium |
| THW |  | uncultured_forest_soil_bacterium |
| THW |  | uncultured_actinobacterium_Nocardioides |
| THW |  | Rhodococcus_erythropolis |
| THW |  | Rhodococcus_tukisamuensis |
| THW |  | uncultured_Actinoallomurus_sp._Acidothermus |
| THW |  | uncultured_Thermomonosporaceae_bacterium_Acidothermus |
| THW |  | uncultured_bacterium_Acidothermus |
| THW |  | Actinoallomurus_spadix |
| THW |  | uncultured_bacterium_Actinomadura |
| THW |  | Thermomonospora_chromogena |
| THW |  | Streptomyces_albus |
| THW |  | Streptomyces_canarius |
| THW |  | Streptomyces_tsusimaensis |
| THW |  | uncultured_bacterium_Streptomyces |
| THW |  | uncultured_bacterium_Tetrasphaera |
| THW |  | uncultured_bacterium_2 |
| THW |  | uncultured_bacterium_Pseudoclavibacter |
| THW |  | Micrococcus_luteus |
| THW |  | Micrococcus_luteus_NCTC_2665 |
| THW |  | Pseudarthrobacter_oxydans |
| THW |  | Pseudarthrobacter_polychromogenes |
| THW | Bacteroidetes | uncultured_soil_bacterium_Chryseolinea |
| THW | Bacteroidetes | Hymenobacter_aerophilus |
| THW | Bacteroidetes | Dyadobacter_hamtensis |
| THW | Bacteroidetes | uncultured_Bacteroidetes_bacterium_Dyadobacter* |
| THW | Bacteroidetes | uncultured_bacterium_3* |
| THW | Bacteroidetes | uncultured_bacterium_Chitinophaga |
| THW | Bacteroidetes | uncultured_bacterium_Flavisolibacter |
| THW | Bacteroidetes | uncultured_Chitinophagaceae_bacterium |
| THW | Bacteroidetes | Sphingobacterium_anhuiense |
| THW | Bacteroidetes | Pedobacter_jejuensis |
| THW | Bacteroidetes | Pedobacter_kyungheensis |
| THW | Bacteroidetes | Pedobacter_steynii |
| THW | Bacteroidetes | uncultured_Bacteroidetes_bacterium_Pedobacter* |
| THW | Bacteroidetes | Chryseobacterium_balustinum |
| THW | Bacteroidetes | Chryseobacterium_joostei |
| THW | Bacteroidetes | Flavobacterium_chungangense |
| THW | Bacteroidetes | Flavobacterium_cutihirudinis |
| THW | Bacteroidetes | Flavobacterium_johnsoniae |
| THW | Bacteroidetes | Flavobacterium_piscis |
| THW | Bacteroidetes | Flavobacterium_reichenbachii |
| THW | Bacteroidetes | Flavobacterium_saccharophilum |
| THW | Bacteroidetes | Flavobacterium_sp._ABG |
| THW | Bacteroidetes | uncultured_Flavobacteriaceae_bacterium* |
| THW | Proteobacteria | uncultured_bacterium_ARKDMS-49 |
| THW | Proteobacteria | uncultured_bacterium_ARKICE-90* |
| THW | Proteobacteria | uncultured_bacterium_SPOTSOCT00m83 |
| THW | Proteobacteria | uncultured_bacterium_Elev-16S-509 |
| THW | Proteobacteria | uncultured_marine_bacterium_Elev-16S-509 |
| THW | Proteobacteria | uncultured_Syntrophobacterales_bacterium_H16 |
| THW | Proteobacteria | uncultured_bacterium_H16 |
| THW | Proteobacteria | uncultured_bacterium_BIrii41 |
| THW | Proteobacteria | uncultured_bacterium_Haliangium |
| THW | Proteobacteria | uncultured_bacterium_MSB-4B10 |
| THW | Proteobacteria | uncultured_bacterium_Phaselicystis |
| THW | Proteobacteria | Brevundimonas_vesicularis |
| THW | Proteobacteria | uncultured_alpha_proteobacterium_Brevundimonas |
| THW | Proteobacteria | uncultured_proteobacterium_Brevundimonas* |
| THW | Proteobacteria | uncultured_bacterium_Tardiphaga* |
| THW | Proteobacteria | Methylobacterium_mesophilicum |
| THW | Proteobacteria | Rhizobium_sp._CCBAU_03068 |
| THW | Proteobacteria | Rhizobium_etli |
| THW | Proteobacteria | uncultured_bacterium_Rhizobium |
| THW | Proteobacteria | uncultured_bacterium_Candidatus_Methylopumilus* |
| THW | Proteobacteria | uncultured_proteobacterium_SC-I-84 |
| THW | Proteobacteria | uncultured_bacterium_UCT_N117 |
| THW | Proteobacteria | uncultured_Rhodocyclaceae_bacterium_070125-BRIC7-5 |
| THW | Proteobacteria | uncultured_bacterium_070125-BRIC7-5 |
| THW | Proteobacteria | uncultured_bacterium_4 |
| THW | Proteobacteria | Arachis_ipaensis |
| THW | Proteobacteria | uncultured_bacterium_Thauera |
| THW | Proteobacteria | uncultured_bacterium_Alicycliphilus* |
| THW | Proteobacteria | uncultured_bacterium_Limnohabitans* |
| THW | Proteobacteria | uncultured_bacterium_Variovorax |
| THW | Proteobacteria | uncultured_Comamonadaceae_bacterium |
| THW | Proteobacteria | uncultured_bacterium_5* |
| THW | Proteobacteria | uncultured_bacterium_Lysobacter* |
| THW | Proteobacteria | uncultured_bacterium_Rhodanobacter |
| THW | Proteobacteria | Stenotrophomonas_maltophilia |
| THW | Proteobacteria | Acinetobacter_indicus |
| THW | Proteobacteria | Acinetobacter_sp._A47 |
| THW | Proteobacteria | Pseudomonas_chlororaphis_subsp._piscium |
| THW | Proteobacteria | Pseudomonas_fluorescens |
| THW | Proteobacteria | Pseudomonas_graminis |
| THW | Proteobacteria | Pseudomonas_helmanticensis |
| THW | Proteobacteria | Pseudomonas_hunanensis |
| THW | Proteobacteria | Pseudomonas_reactans |
| THW | Proteobacteria | Pseudomonas_sp._UK4 |
| THW | Proteobacteria | Pseudomonas_syringae_pv._actinidiae_ICMP_18807 |
| THW | Proteobacteria | uncultured_gamma_proteobacterium_Pseudomonas* |
